# First detection and complete genome analysis of porcine circovirus‐like virus P1 and porcine circovirus‐2 in yak in China

**DOI:** 10.1002/vms3.911

**Published:** 2022-09-01

**Authors:** Jiaping Zhu, Qi Xiao, Libin Wen, Lihong Yin, Fengxi Zhang, Tianjiao Li, Zelang Banma, Kongwang He, Sizhu Suolang

**Affiliations:** ^1^ Institute of Veterinary Medicine Jiangsu Academy of Agricultural Sciences, Key Laboratory of Veterinary Biological Engineering and Technology Ministry of Agriculture and Rural Affairs Nanjing China; ^2^ College of Animal Science Tibet Agricultural and Animal Husbandry University, Provincial Key Laboratory of Tibet Plateau Animal Epidemic Disease Research LinZhi China; ^3^ Jiangsu Co‐innovation Center for Prevention and Control of Important Animal Infectious Diseases and Zoonoses Yangzhou University Yangzhou China; ^4^ Jiangsu Key Laboratory for Food Quality and Safety—State Key Laboratory Cultivation Base of Ministry of Science and Technology Nanjing China

**Keywords:** phylogeny, porcine circovirus‐like virus P1, porcine circovirus‐2, sequencing, yak

## Abstract

Porcine circovirus‐like virus P1, like porcine circovirus type 2 (PCV2), is a potential pathogen of post‐weaning multisystemic wasting syndrome in swine. Yaks are a valuable species and an iconic symbol of the Tibet Plateau which is the highest and largest plateau in the world. In this study, a total of 105 yak diarrheal samples, collected from 13 farms in Linzhi in the Tibet Plateau from January 2019 to December 2021, that were screened for P1 and PCV2 by polymerase chain reaction, 10.48% (*n* = 11) were positive for P1, 4.76% (*n* = 5) for PCV2, and 5.71% (*n* = 6) were positive for coinfection of P1 and PCV2. In addition, the whole genomes of eight P1 strains and eight PCV2 strains were sequenced. Alignment of deduced amino acid sequences of P1 ORF1 and PCV2 ORF2 gene revealed that ON012566 had one unique amino acid mutation at residues 137 (T to P). This mutation has important implication for the study of virus virulence, tissue tropism, and immune response. Phylogenetic analysis shows that the yak‐origin P1 strains in this study with cattle‐origin P1 reference strains were grouped into one cluster. The yak‐origin PCV2 (ON012566) and a buffalo‐origin PCV2 (KM116514) reference strain clustered in the same branch in the PCV2b regions. Meanwhile, the remaining PCV2 strains and buffalo‐origin PCV2 reference strain (ON012565) clustered in the PCV2d regions. To summarize, to our knowledge, this is the first report on the molecular prevalence and genome characteristics of P1 and PCV2 in yaks in the world and will contribute to further study of the molecular epidemiology, source, and evolution of P1 and PCV2 strains.

## Graphical abstract

1. Porcine circovirus‐like virus P1, like Porcine circovirus type 2, is a potential pathogen of post‐weaning multisystemic wasting syndrome (PMWS) in swine.
2. P1 or PCV2 has a risk of cross‐species transmission. P1 has high homology with PCV1 and PCV2 and both PCV1 and PCV2 can be detected in human samples. For the sake of public health and safety, we should attach importance to the research of cross‐species transmission of P1 and PCV2.
3. Yaks are a valuable species and an iconic symbol of the Tibet Plateau, which is the highest and largest plateau in the world. The research on the genetic evolution of the virus in such a special environment is of great significance.
4. To the best of our knowledge, this is the first report of circulation of PCV2 and P1 among yaks in the world.

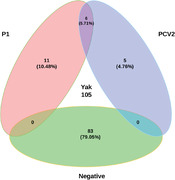

## 1 INTRODUCTION

Porcine circoviruses (PCVs) are small, non‐enveloped DNA viruses and are members of circovirus genus of circovirus family (Mankertz et al., 2004). Generally, the PCVs genomes are single‐stranded circular DNA, and the genome size varies from 1700 to 2000 bp. At present, PCV can be classified into four types: porcine circovirus type 1 (PCV1), PCV2, PCV3, and PCV4. PCV1 was originally discovered in 1974 and is considered to have no pathogenicity in pigs (Tischer et al., 1974). In 1996, a distinct virus was recovered from post‐weaning multisystemic wasting syndrome (PMWS), and it is genetically and antigenically distinct from PCV1, named porcine circovirus 2 (Ellis et al., 1998). Recently, in the United States, PCV3 was identified in pigs with cardiac and multisystemic inflammation, and with clinical signs of porcine dermatitis and nephropathy syndrome (PDNS) (Phan et al., 2016). In 2019, a novel virus was identified in pigs with a severe clinical disease in China named porcine circovirus 4 (H. Zhang et al., 2019). PCV2 has an ambisense genome organization encoding two major open reading frames (ORFs): ORF1 encoding the 35.7‐kDa replication‐associated protein (Rep) and ORF2 encoding the 27.8‐kDa viral capsid protein (Cap) on opposite strands of the double stranded DNA replicative intermediate (Mankertz et al., 1998; Nawagitgul et al., 2000). The clinical forms of PCV2 infection are PMWS, PDNS, porcine respiratory disease complex, reproductive failure, enteric, necrotizing lymphadenitis, exudative dermatitis, and congenital tremor (Allan & Ellis, 2000). Therefore, the diseases associated with PCV2 infection have a serious economic impact on the swine industry worldwide.

Porcine circovirus‐like virus P1 is an emerging causative pathogen of PMWS (Wen, Mao, Jiao, et al., 2017). The genome of P1 consists of a covalently closed, single‐stranded, circular molecule of only 648 nucleotides which is highly homologous to the partial genome sequences of PCV2 (AF381175), but different in only 16 consecutive nucleotides (CGTTACTAGTGGATCC) (D. Zhang et al., 2018). P1 shares 64.39% identity with the consensus region of PCV1 (GU722334) (Wen et al., 2012). The P1 genome encodes three major ORFs: ORF1 and ORF2 are encoded on the antisense strand, whereas ORF3 is encoded on the sense strand. P1 ORF1 encodes the 27.8‐kDa viral capsid protein (Cap) and has extensive homology to the N‐terminal domain of the PCV2 Cap protein (Wen et al., 2014). Like PCV2, P1 can reproduce many clinical symptoms, such as pallor of the skin and diarrhoea (Wen et al., 2012). In addition, P1 has been prevalent in China since its discovery (Wen, Jiao, et al., 2017).

Recently, the P1 virus was found in various animals, such as dogs, pigs, cattle, cats, rabbits, and goats (Wen, Mao, Fan, et al., 2017; Wen et al., 2020). In 2007, the presence of PCV1 in wild boars in Germany was described using serological methods (Cságola et al., 2008). Moreover, in 1995, for the first time, Tischer et al. (1995) confirmed the presence of PCV1 antibodies in cattle in Germany. For PCV2 in non‐porcine hosts, there was evidence in previous studies. In Canada, a PCV2 nucleotide was identified in cattle with respiratory diseases and aborted bovine fetuses (Nayar et al., 1999). In the United States and China, PCV2 was frequently detected in beef from supermarkets, beef stalls, and goat samples (Li et al., 2011; Zhai et al., 2016; W. Zhang et al., 2014). In addition, PCV2 was suggested as a potential causal factor of haemorrhagic diathesis, which can cause moderate clinical signs, viraemia, and seroconversion after infecting calves (Halami et al., 2013, 2014; Kappe et al., 2010). Stunning, PCV1, and PCV2 were detected in human samples, respectively (Bernstein et al., 2003; Esona et al., 2014; Li et al., 2010). It can pose a significant threat to public health and security.

The yak (*Bos grunniens*) belongs to the genus Bovidae and is a unique species of long‐haired cattle, with a population of about 15 million yaks living on the Tibetan plateau over 4000 m above the sea level, accounting for more than 90% of the total yak population in the world (Cui et al., 2020; Yang et al., 2017; Q. Zhang et al., 2016). Yak has high adaptability to harsh environments of high elevation, strong ultraviolet, low oxygen, and low temperatures, and is capable of providing meat, milk, skins, transport, and fuel (faeces), making it an indispensable animal for the local people in the Tibet Plateau (Wu et al., 2020). However, diarrhoea in yak is a common disease, and many viruses such as bovine viral diarrhoea virus and Nebovirus have been identified as important diarrhoea‐causing viruses circulating in the yak. Interestingly, P1 or PCV2 can cause diarrhoea in swine (Allan & Ellis, 2000; Wen et al., 2012). However, the information regarding the prevalence characteristics of P1 or PCV2 in yaks remains unclear. Here, we aimed to investigate the prevalence and molecular characteristics of P1 or PCV2 in yaks in Linzhi, Tibet. To the best of our knowledge, this is the first report of the circulation of P1 and PCV2 among yaks in the world.

## 2 MATERIALS AND METHODS

From January 2019 to December 2021, 105 faeces samples were collected from 105 diarrhoeic yaks at 13 yak farms in the city of Linzhi (Tibet Autonomous Region, China). Samples were stored at −80°C before analysis. To get reliable results, samples were collected in eight different places at each farm. All faecal samples were fresh, taken immediately after defecation.

Total DNA was extracted from the samples using the AllPure DNA/RNA kit (Magen, Guangzhou, China) according to the manufacturer's instructions, and polymerase chain reaction (PCR) was carried out using the high‐fidelity thermostable DNA polymerase (Vazyme, Nanjing, China). PCR amplification was performed as previously described (Wen, Mao, Fan, et al., 2017; Zhai et al., 2016). The PCR amplicons were purified with a Gel Extraction Kit (Omega, Beijing, China), ligated into the pMD18‐T vector (Takara, Dalian, China) and transferred *Escherichia coli* DH5α competent cells (TransGen, Beijing, China) for sequencing, and positive clones were sequenced by TsingKe Biotech (Nanjing, China) as described. Every sequence obtained was derived from at least three independent determinations. In addition, all samples were screened for PCV3 (Palinski et al., 2016) and PCV4 (H. Zhang et al., 2019) by conventional PCR using reference primers.

The complete genome sequences were compared using the Clustal W function of the MegAlign program in DNAstar bioinformatics software (version 7.01). A phylogenetic tree of the complete genome sequences was constructed by the neighbour‐joining method in Molecular Evolutionary Genetics Analysis (MEGA) software (version 11.0) with a *p*‐distance model, using 1000 bootstrap replicates. BioEdit tools (version 7.2) were used for sequence alignment and mutation analysis. We used 28 representative strains (P1, *n* = 13; PCV2, *n* = 15) for phylogenetic analysis, including cattle‐origin P1 strains, rabbit‐origin P1 strains, goat‐origin P1 strains, pig‐origin P1 strains, buffalo‐origin PCV2 strains, pig‐origin PCV2 strains, and human‐origin PCV2 strains.

## 3 RESULTS AND DISCUSSION

**FIGURE 1:**
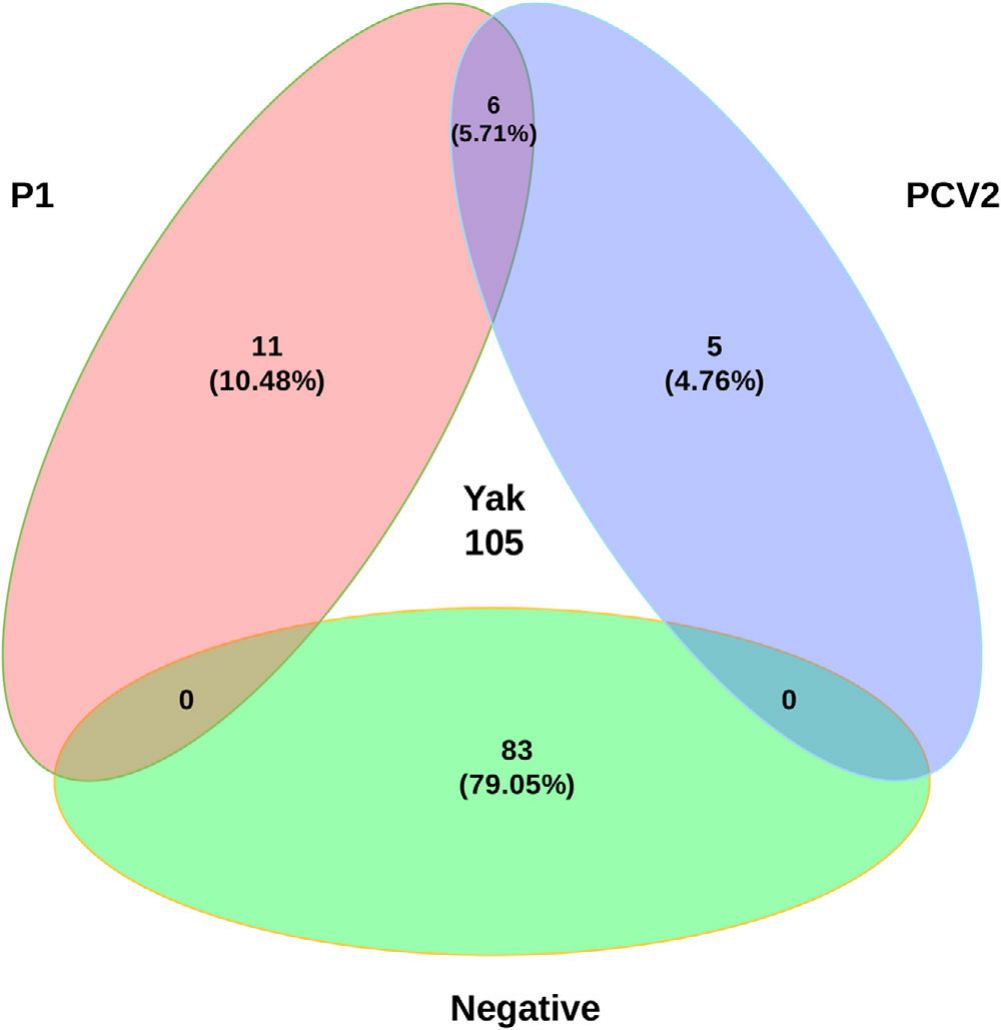
P1‐positive and porcine circovirus type 2 (PCV2)‐positives rate in yaks sampled in Linzhi from 2019 to 2021. All 105 clinical samples form yaks were screened for P1 and PCV2 by PCR, 10.48% (n = 11) were positive for P1 (red area), 4.76% (n = 5) for PCV2 (blue area) and 5.71% (n = 6) were positive for co‐infection of P1 and PCV2 (the intersecting part).

All 105 clinical samples from yaks were screened for P1 and PCV2 by PCR, and the positivity rate was 10.48% (*n* = 11) for P1, 4.76% (*n* = 5) for PCV2, and 5.71% (*n* = 6) for coinfection of P1 and PCV2 (Figure 1). Moreover, PCV3 and PCV4 were also detected in these samples (data not shown). We detected P1 and PCV2 in yak diarrhoeic samples for the first time, which provides the possibility that P1 and PCV2 can be transmitted through yak faeces and have important implications for further investigation of whether P1 and PCV2 cause diarrhoea in yaks. In previous studies, there was a risk of cross‐species transmission of P1 or PCV2. In addition, yaks are free‐grazing domestic animals in the Tibet Plateau, with a large living area where Tibetan pigs and many wild ruminants are found. Therefore, in these regions, P1 or PCV2 has a risk of cross‐species transmission that will bring great challenges to virus prevention and control.

**FIGURE 2:**
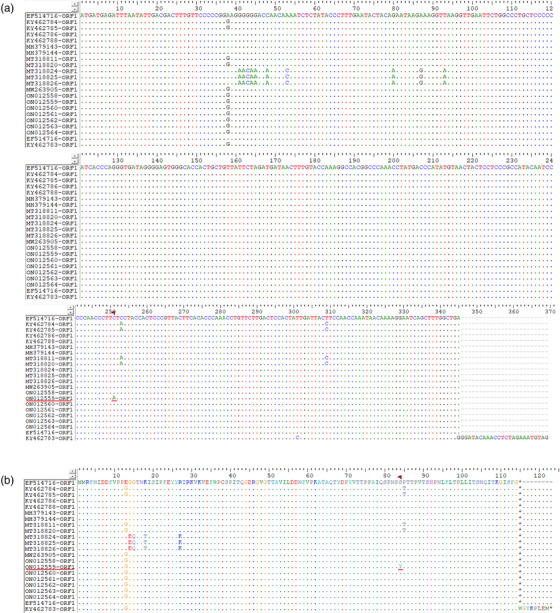
Analysis of nucleic acid sequence and amino acid sequence of P1 ORF1. The nucleotide sequence of P1 ORF1 (a) and the deduced amino acid sequence (b) were compared with the previously reported sequences and amino acid sequences of P1 ORF1 genes, respectively. This alignment included deduced nucleotide sequences of 8 P1 ORF1 sequences from this study and 13 P1 ORF1 reference sequences from Genbank. All the sequences were aligned in Clustal W and viewed in BioEdit sequence alignment editor tool. Mutant strains and mutant sites are marked with red underlines.

**FIGURE 3:**
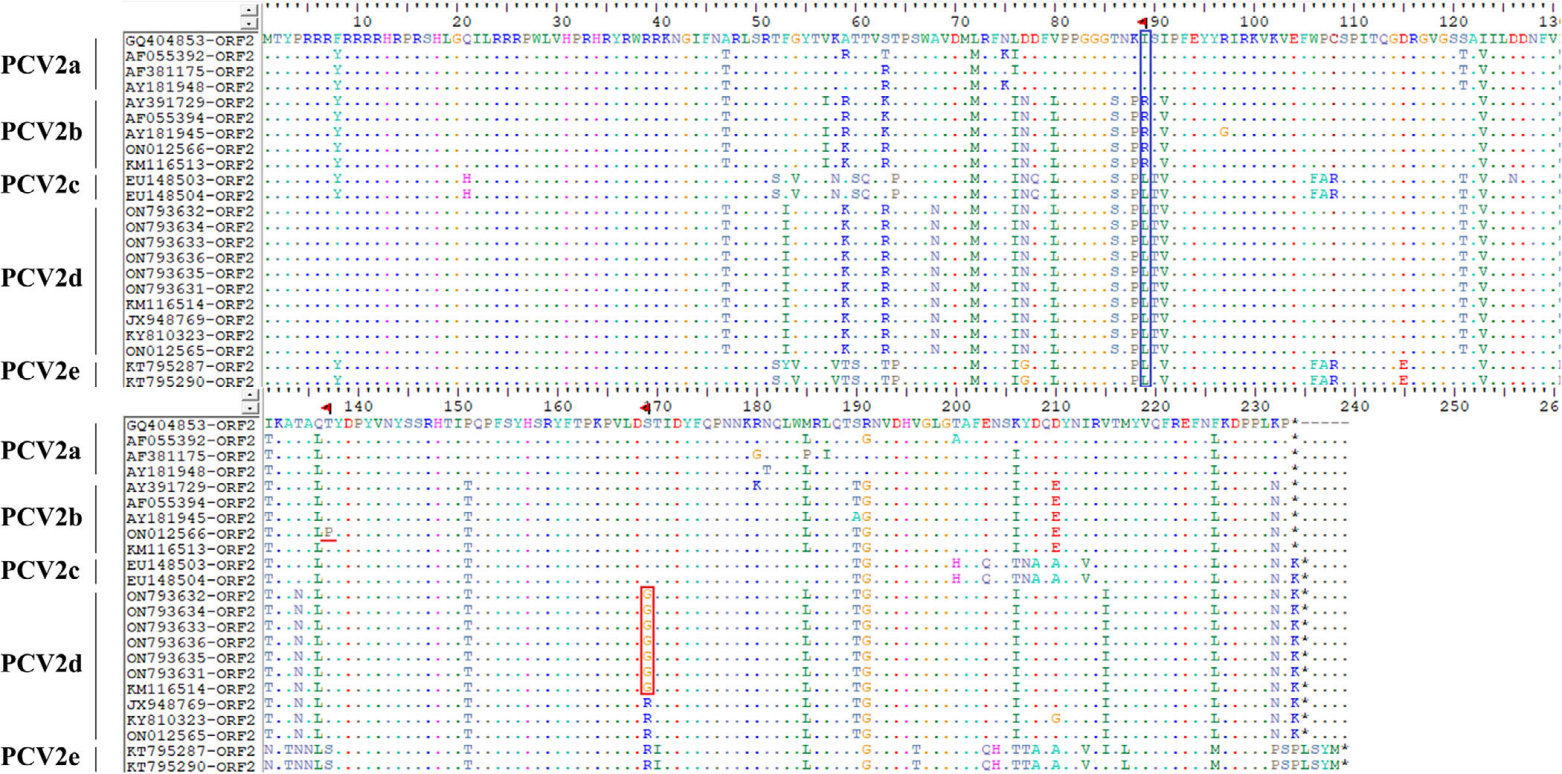
Alignment of deduced amino acid sequences of porcine circovirus type 2 (PCV2) ORF2 gene.This alignment included deduced amino acid sequences of ORF2 from 8 PCV2 sequences from this study and 15 PCV2 reference sequences of different genotypes from Genbank. All the sequences were aligned in Clustal W and viewed in BioEdit sequence alignment editor tool. Mutation sites marked with red underline or red box. Position 89, marked with a blue box, could differentiate PCV2a, PCV2b, and PCV2d, which harbored isoleucine (I), arginine (R), and leucine (L) residues, respectively.

To further study the molecular epidemiology of P1 and PCV2, eight complete yak‐origin P1 and eight complete yak‐origin PCV2 sequences were obtained from P1‐positive and PCV2‐positive clinical samples detected in this study and submitted to the GenBank database (the host, size, genotype, and GenBank accession numbers of these strains are summarized in Table 1). All the eight P1 isolates in this study had a circular genome length of 648 nt and shared 99.2%–100% nucleotide identity with other available P1 genomes in GenBank. Genome sequences of the eight P1 isolates have high nucleotide similarity to two buffalo‐origin reference strains (KY462784 and KY462783). The nucleic acid sequence alignment of the 21 P1 ORF1 (eight isolates from this study and 13 reference strains [Table 1] from different host species) strains showed that LZ‐LL2 (ON012559) had one difference from other P1 sequences: from C to A at position 521 (Figure 2). Meanwhile, amino acid sequence alignments showed that the LZ‐LL2 (ON012559) sequence had one amino acid mutations at residue 84 (S to Y). In this study, the complete genomes of the eight PCV2 strains were obtained, of which one belonged to PCV2b and seven belonged to PCV2d. Four strains of PCV2d (ON793632, ON793633, ON793634, and ON793635) and one strain of PCV2b (ON012566) were coinfected with P1 virus, respectively, and the remaining strains were infected with PCV2 alone. All the eight PCV2 strains had genomes of 1767 bp in length and shared 92.4%–99.9% nucleotide identity with other available PCV2 genomes in GenBank and have high nucleotide similarity to two buffalo‐origin reference strains (KM116513 and KM116514). After analysis, the strains in this study had the same size ORF1 (position from 51 to 995). However, there were distinct differences in ORF2 between ON012566 (702 bp, position from 1734 to 1033) and other strains (705 bp, position from 1734 to 1030). Amino acid sequence alignments of the Cap protein of 23 PCV2 isolates (eight isolates from this study and 15 reference strains from the world in Table 1) showed variability. These variable regions correspond to three antigenic domains (at amino acid positions 65–87, 113–139, and 193–207) (Morozov et al., 1998). Change in amino acid composition at these sites may alter virus characters such as virulence, tissue tropism, and immune response (Saha et al., 2011). The strain ON012566 had one difference at position 137 (T to P) from other reference PCV2‐ORF2 amino acid sequences (Figure 3). Therefore, further study on this special site will be interesting.

**TABLE 1:** P1 and porcine circovirus type 2 (PCV2) sequences used in this study

Strain name	Accession number	Genotype	Size (nt)	Host	Country
**LZ‐LL1**	**ON012558**	**P1**	**648**	**Yak**	**China**
**LZ‐LL2**	**ON012559**	**P1**	**648**	**Yak**	**China**
**LZ‐LL3**	**ON012560**	**P1**	**648**	**Yak**	**China**
**LZ‐LL4‐1**	**ON012561**	**P1**	**648**	**Yak**	**China**
**LZ‐LL4‐2**	**ON012562**	**P1**	**648**	**Yak**	**China**
**LZ‐LL5**	**ON012563**	**P1**	**648**	**Yak**	**China**
**XZ‐SJ**	**ON012564**	**P1**	**648**	**Yak**	**China**
**Tibet‐P1**	**MW263905**	**P1**	**648**	**Yak**	**China**
**Yak‐B2**	**ON012565**	**PCV2**	**1767**	**Yak**	**China**
**Yak‐B6**	**ON012566**	**PCV2**	**1767**	**Yak**	**China**
**Yak‐11**	**ON793631**	**PCV2**	**1767**	**Yak**	**China**
**Yak‐13**	**ON793632**	**PCV2**	**1767**	**Yak**	**China**
**Yak‐15**	**ON793633**	**PCV2**	**1767**	**Yak**	**China**
**Yak‐24**	**ON793634**	**PCV2**	**1767**	**Yak**	**China**
**Yak‐38**	**ON793635**	**PCV2**	**1767**	**Yak**	**China**
**Yak‐77**	**ON793636**	**PCV2**	**1767**	**Yak**	**China**
HB1	EF514716	P1	648	Porcine	China
JSDY	KY462783	P1	648	Cattle	China
JSJN	KY462784	P1	648	Cattle	China
JSZJ	KY462786	P1	648	Rabbit	China
JSLYG	KY462788	P1	648	Goat	China
JSNJ	KY462785	P1	648	Rabbit	China
NJ01	MH379143	P1	648	Dog	China
NJ02	MH379144	P1	648	Dog	China
HeB01	MT318811	P1	648	Dog	China
NJ01	MT318820	P1	648	Cat	China
HeB02	MT318824	P1	648	Cat	China
NJ01	MT318825	P1	648	Cat	China
NJ02	MT318826	P1	648	Cat	China
MN500	GQ404853	PCV2a	1768	Homo sapiens	USA
Porcine circovirus type II	AF055392	PCV2a	1768	Porcine	Canada
BF	AF381175	PCV2a	1768	Porcine	China
SZ	AY181948	PCV2a	1768	Porcine	China
GD‐TS	AY181945	PCV2b	1767	Porcine	China
Buffalo1	KM116514	PCV2b	1767	Buffalo	China
Porcine circovirus type II	AF055394	PCV2b	1767	Porcine	France
NB0301	AY391729	PCV2b	1767	Porcine	China
DK1987PMWSfree	EU148504	PCV2c	1767	Porcine	Denmark
DK1980PMWSfree	EU148503	PCV2c	1767	Porcine	Denmark
CZ1005	JX948769	PCV2d	1767	Porcine	China
HID5707	KY810323	PCV2d	1767	Porcine	China
Buffalo2	KM116514	PCV2d	1767	Buffalo	China
MEX/41238/2014	KT795287	PCV2e	1777	Porcine	Mexico
USA/45358/2015	KT795290	PCV2e	1777	Porcine	USA

*Note*: Ten genome sequences obtained in this study are shown in boldface.

**FIGURE 4:**
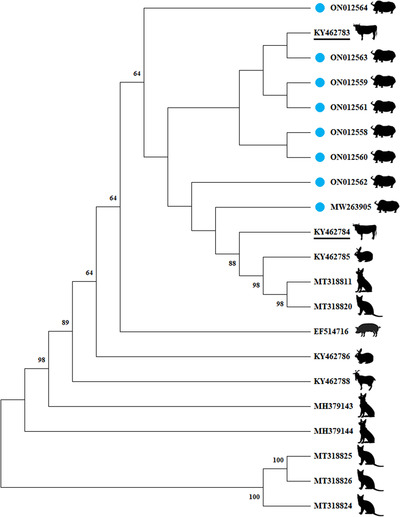
Phylogenetic analysis of eight yak‐origin P1 strains in this study and other reference strains. The phylogenetic tree was constructed by the neighbor‐joining method using MEGA 11.0 software. Bootstrap replications were set by 1000. Note: Eight yak‐origin P1 strains in this study labeled by blue circles and two cattle‐origin P1 reference sequences were labeled using underlines.

**FIGURE 5:**
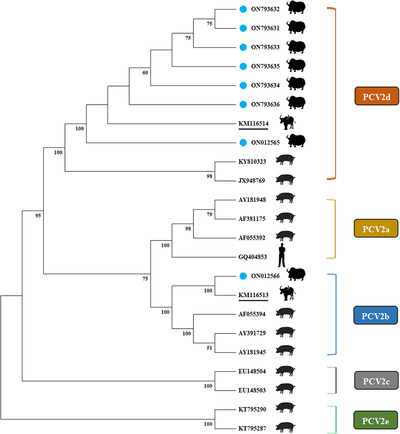
Phylogenetic analysis of eight yak‐origin porcine circovirus type 2 (PCV2) strains in this study and other reference strains. The phylogenetic tree was constructed by the neighbor‐joining method using MEGA 11.0 software. Bootstrap replications were set by 1000. Note: Eight yak‐origin PCV2 strains in this study labeled by blue circles and two buffalo‐origin PCV2 reference sequences were labeled using underlines.

A corresponding phylogenetic tree (including 13 reference P1 sequences and eight P1 sequences from this study) was constructed based on the full‐length sequence. Phylogenetic analysis suggested that LZ‐LL2 (ON012559), LZ‐LL4‐1 (ON012561), LZ‐LL4‐2 (ON012562), and Tibet‐P1 (MW263905) were located in a separate branch, and LZ‐LL1 (ON012558), LZ‐LL3 (ON012560), LZ‐LL5 (ON012563), and XZ‐SJ (ON012564) were clustered with KY462783 (cattle‐origin P1 strain) in a small branch of the phylogenetic tree. Interestingly, all P1 sequences in this study are to be clustered in a large branch of the phylogenetic tree and similar with two cattle‐origin reference strains (KY462784 and KY462783), and the eight P1 sequences were closely related to each other (Figure 4). The results of PCV2 showed that ON012565, ON793631, ON793632, ON793633, ON793634, ON793635, and ON793636 belonged to genotype PCV2d and were clustered in the same branches of the phylogenetic tree with buffalo‐origin PCV2d reference strains (KM116514) (Figure 5). ON012566 belonged to PCV2b, whose nucleotide was similar with buffalo‐origin PCV2b reference strain (KM116513) and were clustered in the same branch of the phylogenetic tree (Figure 5). Furthermore, we could easily differentiate PCV2a, PCV2b, and PCV2d by amino acid position 89 in ORF2, which harboured isoleucine (I), arginine (R), and leucine (L) residues, respectively (Cheung et al., 2007). At amino acid position 89 in ORF2, ON012565, ON793631, ON793632, ON793633, ON793634, ON793635, and ON793636 are leucine and ON012566 is arginine We could also distinguish different genotypes (Figure 3). Meanwhile, compared with other reference PCV2d strains, ON793631, ON793632, ON793633, ON793634, ON793635, and ON793636 had one amino acid mutations at residues 169 (S to G). These results provide a basis for structural and functional studies of the P1 and PCV2.

The Tibet Plateau, with an average altitude of 4000 m, thin air, strong ultraviolet radiation, and large temperature difference throughout the year, has bred many special species, including yaks and Tibetan pigs, and endowed these species with strong tolerance and cold resistance (Ma et al., 2019; Wu et al., 2020; Yang et al., 2017). Research on the genetic evolution of the virus in such a special environment is of great significance. In previous studies, there is a risk of cross‐species transmission of P1 or PCV2 (Esona et al., 2014; Wen, Mao, Fan, et al., 2017). In this research, genetic phylogenetic tree analysis showed that yak‐origin P1 was located in several branches and distributed relatively intensively, indicating that yak‐origin P1 had a certain regional specificity and genetic diversity. In addition, the phylogenetic tree analysis showed that the yak‐origin P1 was closest to P1 isolates from cattle‐origin and far away from domestic pigs, rabbits, goats, and other species, indicating that the yak‐origin P1 virus had a certain species specificity. In previous studies, PCV2‐infected calves demonstrate that host susceptibility of PCV2 is not solely restricted to pigs (Halami et al., 2014). In this study, the phylogenetic tree analysis showed the yak‐origin PCV2 strains were in separate branches and far away from domestic pigs and humans, indicating that the yak‐origin PCV2 has certain species specificity and genetic diversity (Figure 5). Significantly, yak‐origin P1 and yak‐origin PCV2 are close to cattle‐origin P1 and buffalo‐origin PCV2, respectively. Yak, buffalo, and cattle all belong to the genus Bos in the family Bovidae. In viruses, host adaptability may be an evolutionary process linked to the balance between genetic variation and selection. In addition, this study found that there are still some genetic differences between yak‐origin P1 and cattle‐origin P1 or yak‐origin PCV2 and buffalo‐origin PCV2, indicating that yak‐origin P1 and yak‐origin PCV2 strains have unique evolutionary characteristics. Therefore, the unique evolution of P1 and PCV2 in yaks may be related to the special geographical environment (such as the high‐altitude, strong ultraviolet, low oxygen, low temperature, and low atmospheric pressure) of the Tibet Plateau where the yak lives, as well as to the characteristics of the host species. Yaks have large living areas where there are many wild animals. Therefore, P1 and PCV2 from yaks have the potential for transboundary and cross‐species transmissions. The candidate vaccine based on those prevailing viruses paves the way to combat this emerging pathogen. Surprisingly, both PCV1 and PCV2 can be detected in human samples. In addition, P1 has high homology with PCV1 and PCV2, suggesting that P1 is closely related to PCV1 and PCV2. However, at present, there are no reports of detection of the P1 virus in human samples. For the sake of public health and safety, we should pay attention to the importance of the research on cross‐species transmission of P1 and PCV2.

In conclusion, to our knowledge, this is the first report on the molecular prevalence and genome characteristics of P1 and PCV2 in yaks in the world. This report investigated the circulation of P1 or PCV2 in yaks in China, showing genetic diversity. However, further studies will be required to ascertain whether P1 or PCV2 causes diarrhoea in yaks. This finding will contribute to the diagnosis and prevention of yak diarrhoea in China, and it will contribute to further study of the molecular epidemiology, source, and evolution of P1 and PCV2 strains in yaks.

## AUTHOR CONTRIBUTIONS

*Conceptualization, data curation, investigation, methodology, writing—original draft, and writing—review and editing*: Jiaping Zhu. *Data curation, methodology, and resources*: Qi Xiao. *Data curation, formal analysis, and supervision*: Libin Wen. *Data curation and validation*: Lihong Yin. *Data curation*: Fengxi Zhang. *Investigation*: Tianjiao Li and Zelang Banma. *Funding acquisition, project administration, and supervision*: Kongwang He. *Funding acquisition, project administration, and supervision*: Sizhu Suolang.

## CONFLICT OF INTEREST

The authors declare no conflict of interest.

### ETHICS STATEMENT

The study did not involve animal experiments, and there were no animal ethics problems.

### PEER REVIEW

The peer review history for this article is available at https://publons.com/publon/10.1002/vms3.911.

## Data Availability

The genome sequences resulting from this study were deposited into the GenBank database with accession numbers MW263905, ON012558‐ON012566, and ON793631‐ON793636.

## References

[vms3911-bib-0001] Allan, G. M. , & Ellis, J. A. (2000). Porcine circoviruses: A review. Journal of Veterinary Diagnostic Investigation, 12(1), 3–14. 10.1177/104063870001200102 10690769

[vms3911-bib-0002] Bernstein, C. N. , Nayar, G. , Hamel, A. , & Blanchard, J. F. (2003). Study of animal‐borne infections in the mucosas of patients with inflammatory bowel disease and population‐based controls. Journal of Clinical Microbiology, 41(11), 4986–90. 10.1128/JCM.41.11.4986-4990.2003 14605128 PMC262476

[vms3911-bib-0003] Cheung, A. K. , Lager, K. M. , Kohutyuk, O. I. , Vincent, A. L. , Henry, S. C. , Baker, R. B. , Rowland, R. R. , & Dunham, A. G. (2007). Detection of two porcine circovirus type 2 genotypic groups in United States swine herds. Archives of Virology, 152(5), 1035–1044. 10.1007/s00705-006-0909-6 17219018 PMC7086833

[vms3911-bib-0004] Cui, P. , Feng, L. , Zhang, L. , He, J. , & Yang, X. (2020). Antimicrobial resistance, virulence genes, and biofilm formation capacity among Enterococcus species from yaks in Aba Tibetan autonomous prefecture, China. Frontiers in Microbiology, 11, 1250. 10.3389/fmicb.2020.01250 32595625 PMC7304059

[vms3911-bib-0005] Cságola, A. , Kiss, I. , & Tuboly, T. (2008). Detection and analysis of porcine circovirus type 1 in Hungarian wild boars: Short communication. Acta Veterinaria Hungarica, 56(1), 139–44. 10.1556/AVet.56.2008.1.15 18401965

[vms3911-bib-0006] Ellis, J. , Hassard, L. , Clark, E. , Harding, J. , Allan, G. , Willson, P. , Strokappe, J. , Martin, K. , McNeilly, F. , Meehan, B. , Todd, D. , & Haines, D. (1998). Isolation of circovirus from lesions of pigs with postweaning multisystemic wasting syndrome. Canadian Veterinary Journal, 39(1), 44–51. 10.2307/1592601 PMC1539838 9442952

[vms3911-bib-0007] Esona, M. D. , Mijatovic‐Rustempasic, S. , Yen, C. , Parashar, U. D. , Gentsch, J. R. , Bowen, M. D. , & LaRussa, P. (2014). Detection of PCV‐2 DNA in stool samples from infants vaccinated with RotaTeq®. Human Vaccines & Immunotherapeutics, 10(1), 25–32. 10.4161/hv.26731. 10.4161/hv.26731 24104203 PMC4181015

[vms3911-bib-0008] Halami, M. Y. , Hermann, M. , Jens, B. , & Vahlenkamp, T. W. (2013). Whole‐genome sequences of two strains of porcine circovirus 2 isolated from calves in Germany. Genome Announcements, 2(1), e01150–13. 10.1128/genomeA.e01150-13 PMC3886960 24407647

[vms3911-bib-0009] Halami, M. Y. , Freick, M. , Shehata, A. A. , Müller, H. , & Vahlenkamp, T. W. (2014). Susceptibility of calves to porcine circovirus‐2 (PCV2). Veterinary Microbiology, 173(1–2), 125–131. 10.1016/j.vetmic.2014.06.022 25085519

[vms3911-bib-0010] Kappe, E. C. , Halami, M. Y. , Schade, B. , Alex, M. , & Hermann, M. (2010). Bone marrow depletion with haemorrhagic diathesis in calves in Germany: Characterization of the disease and preliminary investigations on its aetiology. Berliner Und Munchener Tierarztliche Wochenschrift, 123(1–2), 31–41. 10.2376/0005-9366-123-31 20135908

[vms3911-bib-0011] Li, L. , Kapoor, A. , Slikas, B. , Bamidele, O. S. , Wang, C. , Shaukat, S. , Masroor, M. A. , Wilson, M. L. , Ndjango, J. B. , Peeters, M. , Gross‐Camp, N. D. , Muller, M. N. , Hahn, B. H. , Wolfe, N. D. , Triki, H. , Bartkus, J. , Zaidi, S. Z. , & Delwart, E. (2010). Multiple diverse circoviruses infect farm animals and are commonly found in human and chimpanzee feces. Journal of Virology, 84, 1674–82. 10.1128/JVI.02109-09 20007276 PMC2812408

[vms3911-bib-0012] Li, L. , Shan, T. , Soji, O. B. , Alam, M. M. , Kunz, T. H. , & Zaidi, S. Z. , & Delwart, E. (2011). Possible cross‐species transmission of circoviruses and cycloviruses among farm animals. Journal of General Virology, 92(4), 768–772. 10.1099/vir.0.028704-0 21177928 PMC3133700

[vms3911-bib-0013] Ma, Y. F. , Han, X. M. , Huang, C. P. , Zhong, L. , & Zhang, Y. P. (2019). Population genomics analysis revealed origin and high‐altitude adaptation of Tibetan pigs. Scientific Reports, 9(1), 11463. 10.1038/s41598-019-47711-6 31391504 PMC6685962

[vms3911-bib-0014] Mankertz, A. , Aliskan, R. , Hattermann, K. , Hillenbrand, B. , Kurzendoerfer, P. , Mueller, B. , Schmitt, C. , Steinfeldt, T. , & Finsterbusch, T. (2004). Molecular biology of porcine circovirus: Analyses of gene expression and viral replication. Veterinary Microbiology, 98(2), 81–88. 10.1016/j.vetmic.2003.10.014 14741119

[vms3911-bib-0015] Mankertz, A. , Mankertz, J. , Wolf, K. , & Buhk, H. J. (1998). Identification of a protein essential for replication of porcine circovirus. Journal of General Virology, 79(2), 381–384. 10.1099/0022-1317-79-2-381 9472624

[vms3911-bib-0016] Morozov, I. , Sirinarumitr, T. , Sorden, S. D. , Halbur, P. G. , & Paul, P. S. (1998). Detection of a novel strain of porcine circovirus in pigs with postweaning multisystemic wasting syndrome. Journal of Clinical Microbiology, 121(9), 2535–2541. 10.1128/JCM.36.9.2535-2541.1998 PMC105158 9705388

[vms3911-bib-0017] Nawagitgul, P. , Morozov, I. , Bolin, S. R. , Harms, P. A. , & Paul, P. S. (2000). Open reading frame 2 of porcine circovirus type 2 encodes a major capsid protein. Journal of General Virology, 81(Pt 9), 2281–7. 10.1099/0022-1317-81-9-2281 10950986

[vms3911-bib-0018] Nayar, G. P. S. , Hamel, A. L. , Lin, L. H. , Sachvie, C. , & Spearman, G. (1999). Evidence for circovirus in cattle with respiratory disease and from aborted bovine fetuses. The Canadian Veterinary Journal, 40(4), 277–278. PMC1539691 10200889

[vms3911-bib-0019] Palinski, R. , Piñeyro, P. , Shang, P. , Yuan, F. , Guo, R. , Fang, Y. , Byers, E. , & Hause, B. M. (2016). A novel porcine circovirus distantly related to known circoviruses is associated with porcine dermatitis and nephropathy syndrome and reproductive failure. Journal of Virology, 91(1), e01879–16. 10.1128/JVI.01879-16 27795441 PMC5165205

[vms3911-bib-0020] Phan, T. G. , Giannitti, F. , Rossow, S. , Marthaler, D. , Knutson, T. , Li, L. , Deng, X. , Resende, T. , Vannucci, F. , & Delwart, E. (2016). Detection of a novel circovirus PCV3 in pigs with cardiac and multi‐systemic inflammation. Virology Journal, 13(1), 184. 10.1186/s12985-016-0642-z 27835942 PMC5105309

[vms3911-bib-0021] Saha, D. , Huang, L. , Bussalleu, E. , Lefebvre, D. J. , & Nauwynck, H. J. (2011). Antigenic subtyping and epitopes' competition analysis of porcine circovirus type 2 using monoclonal antibodies. Veterinary Microbiology, 157(1‐2), 13–22. 10.1016/j.vetmic.2011.11.030 22176764

[vms3911-bib-0022] Tischer, I. , Bode, L. , Apodaca, J. , Timm, H. , Peters, D. , Rasch, R. , Pociuli, S. , & Gerike, E. (1995). Presence of antibodies reacting with porcine circovirus in sera of humans, mice, and cattle. Archives of Virology, 140(8), 1427–1439. 10.1007/BF01322669 7544971

[vms3911-bib-0023] Tischer, I. , Rasch, R. , & Tochtermann, G. (1974). Characterization of papovavirus‐ and picornavirus‐like particles in permanent pig kidney cell lines. Zentralbl Bakteriol Orig, 226(2), 153–167. 4151202

[vms3911-bib-0024] Wen, L. , He, K. , Xiao, Q. , Yu, Z. , Mao, A. , Ni, Y. , Zhang, X. , Li, B. , Wang, X. , Guo, R. , Zhou, J. , Lv, L. , & Jiang, J. (2012). A novel porcine circovirus‐like agent P1 is associated with wasting syndromes in pigs. PLoS One, 7(8), e41565. 10.1371/journal.pone.0041565 22936978 PMC3427322

[vms3911-bib-0025] Wen, L. , He, K. , Yu, Z. , Mao, A. , Ni, Y. , Guo, R. , Li, B. , Wang, X. , Zhou, J. , & Lv, L. (2012). Complete genome sequence of a novel porcine circovirus‐like agent. Journal of Virology, 86(1), 639–639. 10.1128/JVI.06469-11 22158848 PMC3255878

[vms3911-bib-0026] Wen, L. , Jiao, F. , Zhang, D. , Li, Y. , Mao, A. , Liu, C. , Xie, J. , & He, K. (2017). Molecular characterization of porcine circovirus‐like virus P1 in eastern China. Infection Genetics & Evolution, 48, 54–57. 10.1016/j.meegid.2016.12.012 27986553

[vms3911-bib-0027] Wen, L. , Mao, A. , Fan, Z. , Li, W. , Xiao, Q. , Liu, Q. , Xie, J. , & He, K. (2017). Porcine circovirus‐like virus P1 in cattle, goats and rabbits in China. Transboundary Emerging Diseases, 65(1), e217–e218. 10.1111/tbed.12716 28941198

[vms3911-bib-0028] Wen, L. , Mao, A. , Jiao, F. , Zhang, D. , Xie, J. , & He, K. (2017). Evidence of porcine circovirus‐like virus P1 in piglets with an unusual congenital tremor. Transboundary Emerging Diseases, 65(2), e501–e504. 10.1111/tbed.12772 29178610

[vms3911-bib-0029] Wen, L. , Mao, A. , Xie, J. , & He, K. (2020). First molecular identification of porcine circovirus‐like agents in dogs and cats in China. Virus Genes, 56(6), 781–784. 10.1007/s11262-020-01796-8 32960437

[vms3911-bib-0030] Wen, L. , Wang, F. , He, K. , Li, B. , Wang, X. , Guo, R. , & Xie, J. (2014). Transcriptional analysis of porcine circovirus‐like virus P1. BMC Veterinary Research, 10, 287. 10.1186/s12917-014-0287-3 25440084 PMC4258304

[vms3911-bib-0031] Wu, S. , Mipam, T. D. , Xu, C. , Zhao, W. , & Zhong, J. (2020). Testis transcriptome profiling identified genes involved in spermatogenic arrest of cattleyak. PLoS One, 15(2), e0229503. 10.1007/s10142-021-00806-8 32092127 PMC7039509

[vms3911-bib-0032] Yang, C. , Liu, J. , Wu, X. , Bao, P. , Long, R. , Guo, X. , Ding, X. , & Yan, P. (2017). The response of gene expression associated with lipid metabolism, fat deposition and fatty acid profile in the *longissimus dorsi* muscle of Gannan yaks to different energy levels of diets. PLoS One, 12(11), e0187604. 10.1371/journal.pone.0187604 29121115 PMC5679530

[vms3911-bib-0033] Zhai, S. L. , Chen, R. A. , Chen, S. N. , Wen, X. H. , Lv, D. H. , Wu, D. C. , Yuan, J. , Huang, Z. , Zhou, X. R. , Luo, M. L. , He, D. S. , & Wei, W. K. (2016). First molecular detection of porcine circovirus type 2 in bovids in China. Virus Genes, 52(1), 160–160. 10.1007/s11262-014-1117-1 26578152

[vms3911-bib-0034] Zhang, D. , He, K. , Wen, L. , & Fan, H. (2018). Protective efficacy of a DNA vaccine encoding capsid protein of porcine circovirus‐like virus P1 against porcine circovirus 2 in mice. Microbiology & Immunology, 62(3), 195–199. 10.1111/1348-0421.12571 29315776

[vms3911-bib-0035] Zhang, H. , Hu, W. , Li, J. , Liu, T. , & Xiao, C. (2019). Novel circovirus species identified in farmed pigs designated as porcine circovirus 4, Hunan province, China. Transboundary and Emerging Diseases, 67(3), 1057–1061. 10.1111/tbed.13446 31823481

[vms3911-bib-0036] Zhang, Q. , Gou, W. , Wang, X. , Zhang, Y. , Ma, J. , & Zhang, H. (2016). Genome resequencing identifies unique adaptations of Tibetan chickens to hypoxia and high‐dose ultraviolet radiation in high‐altitude environments. Genome Biology and Evolution, 8(3), 765–76. 10.1093/gbe/evw032 26907498 PMC4824011

[vms3911-bib-0037] Zhang, W. , Li, L. , Deng, X. , Kapusinszky, B. , & Delwart, E. (2014). What is for dinner? Viral metagenomics of us store bought beef, pork, and chicken. Virology, 468–470, 303–310. 10.1016/j.virol.2014.08.025 PMC4252299 25217712

